# Acute Stress Disorder among 2022 Ukrainian war refugees: a cross-sectional study

**DOI:** 10.3389/fpubh.2024.1280236

**Published:** 2024-03-14

**Authors:** Piotr Kordel, Marcin Rządeczka, Marta Studenna-Skrukwa, Katarzyna Kwiatkowska-Moskalewicz, Olga Goncharenko, Marcin Moskalewicz

**Affiliations:** ^1^Philosophy of Mental Health Unit, Department of Social Sciences and the Humanities, Poznan University of Medical Sciences, Poznań, Poland; ^2^Institute of Philosophy, Marie Curie-Sklodowska University, Lublin, Poland; ^3^IDEAS NCBR, Warsaw, Poland; ^4^Faculty of History, Adam Mickiewicz University, Poznań, Poland; ^5^Department of History, Heidelberg University, Heidelberg, Germany; ^6^Faculty of Social Sciences and Social Technologies, National University of Kyiv-Mohyla Academy, Kyiv, Ukraine; ^7^Psychiatric Clinic, Heidelberg University, Heidelberg, Germany

**Keywords:** refugees, displaced persons, war, stress, trauma, health, Acute Stress Disorder, Ukraine

## Abstract

**Introduction:**

Fleeing from war can be terrifying and result in Acute Stress Disorder (ASD), a mental health condition that can occur in the first month after a traumatic event. The study aimed to identify the prevalence of ASD among Ukrainian refugees and identify its risk factors to create a profile of the most vulnerable refugees.

**Methods:**

This cross-sectional study of 637 Ukrainian war-displaced persons and refugees in 2022 used the Acute Stress Disorder Scale.

**Results:**

The prevalence of ASD among participants was high (93.5%). Several factors increasing the risk of developing ASD in the sample were identified, e.g., witnessing Russian attacks (OR 2.92, 95% CI 1.26–6.78), insufficient financial resources (OR 3.56, 95% CI 1.61–7.91), and feeling of loneliness in the host country (OR 3.07, 95% CI 1.58–8.69). Pre-existing depression and the death of a close person, among others, were found to significantly (*p* < 0.05) exacerbate the ASD symptoms. At the same time, neither age, the distance traveled, time spent on fleeing the country, nor the type of companionship during refuge (escaping alone, with children, pets or the older adults) correlate with the severity of symptoms.

**Conclusion:**

The study shows extreme levels of trauma among Ukrainian war refugees and displaced persons. Knowledge regarding ASD vulnerabilities in the present conflict may facilitate prompt and adequate psychological help. Since ASD can be an antecedent of PTSD and several autoimmune disorders, these results may also serve as a predictor of future challenges for Ukrainian society.

## Introduction

### Russia’s aggression against Ukraine

Russia’s full-scale aggression against Ukraine, which began on February 24th, 2022, resulted in a humanitarian catastrophe comparable to those caused by contemporary armed conflicts in Syria, Iraq, Yemen, Libya, Afghanistan, and Sudan (1). It is the fourth humanitarian disaster to hit Ukrainian society in the last century. Currently, the Ukrainian civilian population is affected by acts that fit the definition of crimes against humanity of the Rome Statute of the International Criminal Court (2) (Article 7), as it was during the Civil War of 1918–1920, the Great Famine of 1932–1933, and World War II. Nearly one-third of Ukrainians have been displaced from their homes, according to UNHCR estimates. Over six million have fled the war, with 90% of displacements being women and children. From 24th February 2022 to 17th April 2023, the Office of the UN High Commissioner for Human Rights (OHCHR) recorded 22,904 civilian casualties in the country: 8,534 killed and 14,370 injured (3). By November 21st, 2023 the number of killed civilians topped 10,000 (4). However, Ukrainian sources reported that in 2022, as a result of Russia’s invasion, 16,502 people were killed (5). According to the UN, the largest numbers of Ukrainian refugees are reported in (excluding Russia) Poland (1,573,267), Germany (1,055,323), and the Czech Republic (501,540). As of 20th March 2023, 8,157,230 Ukrainian refugees were recorded in Europe (6).

Fleeing a war is an acutely stressful experience. The necessity to leave everything, home, belongings, and loved ones within hours or days, combined with the uncertainty of ever coming back, is a traumatizing event. This trauma can be exacerbated by pre-migration and other acutely stressful experiences or potentially traumatic events (PTEs) such as being under shelling (frequently without access to shelters), trapped and unable to meet basic needs, including food, water, and medicines, witnessing war crimes or sex violence and evacuation difficulties. We also cannot forget about additional post-migration factors that could contribute to overall distress: fears for family and friends remaining in Ukraine, the uncertainty of settlement procedures, difficulty accessing healthcare, or challenges in securing stable work and accommodation caused by a large number of refugees, and previous labor migration. These mental health issues can lead to other serious health risks, such as cardiovascular, chronic respiratory, and infectious diseases or diabetes. Moreover, the Ukrainian health care system, strained by the war, faces a lack of funding and limited workforce capacity, worsening the situation for those needing mental assistance [which, to some extent, resembles the case of Iraq (7)], (8, 9). All of this happened during the COVID-19 pandemic, which was an additional source of stress and a considerable challenge for the healthcare system (10, 11).

### Acute and post-traumatic stress

Previous studies of trauma in refugee populations focused mainly on post-traumatic stress disorder (PTSD). This psychiatric condition can occur in people who have experienced or witnessed a traumatic event, such as a natural disaster, a serious accident, a terrorist act, war, rape, or other violent personal assault. PTSD is characterized by a range of symptoms, including re-experiencing trauma through flashbacks or nightmares, avoidance of stimuli associated with the trauma, adverse changes in thoughts and mood, and alterations in arousal and reactivity. These symptoms cause significant distress or impairment in social, occupational, or other important areas of functioning (12). Refugees, having endured severe traumatic events, exhibit markedly high rates of PTSD and complex PTSD (CPTSD), a finding consistent across various studies and cultures (13). Key predictors for PTSD in these groups include torture and a history of multiple traumatic experiences (14). Notably, mental health disorders, including PTSD and depression, are prevalent in up to 30% of refugees, a rate significantly higher than in general populations in Western countries (15, 16). Several epidemiological studies have found a higher prevalence of autoimmune diseases in individuals with PTSD compared to those without. PTSD is associated with chronic stress, which can dysregulate the immune system. This dysregulation can manifest as immune suppression or overactivation, potentially leading to autoimmune disorders. The body’s prolonged stress response in PTSD can alter the functioning of immune cells and the release of cytokines, which are critical in immune system regulation (17, 18).

This study focuses on Acute Stress Disorder (ASD) in the refugee and displaced persons population. ASD is a condition that can develop following a traumatic event and is characterized by symptoms similar to, but generally less severe than, those of PTSD. Symptoms of ASD include intrusive memories, negative mood, dissociation, avoidance of reminders of traumatic events, and increased arousal. These symptoms arise immediately following the trauma but are of a shorter duration, typically lasting from 3 days to up to 1 month after the event. ASD can be a precursor to PTSD, but the relation between these two conditions is complicated and not linear. DSM-5 revised ASD definition emphasizes immediate trauma reactions without implying future PTSD development. This change was influenced by longitudinal studies indicating that not all individuals with eventual PTSD initially meet ASD criteria (19).

This research treats the displacement of Ukrainians caused by the Russian invasion as a primary traumatic experience triggering acute stress reaction with a high risk of developing PTSD, which can be exacerbated by the circumstances in which the displacement was taking place as well as previous traumatic experiences and health conditions of the displaced.

### Aims and scope

The study’s main goal was to assess the incidence of ASD among Ukrainians caused by their displacement and to identify the factors that increase its odds and correlate with increased symptoms. To the best of our knowledge, such research has never been done on a study group consisting of war refugees. Therefore, the results might be helpful for the governments and organizations assisting the displaced persons to assess their needs in terms of psychological support and identifying the most vulnerable, to whom psychological help should be offered first. However, it is worth mentioning that diagnosing both ASD and PTSD in refugees is particularly challenging due to high comorbidity with other mental disorders. Refugees often experience a range of psychological issues, such as depression, anxiety, and substance abuse, which can overlap with the symptoms of ASD and PTSD. This complexity makes it difficult to isolate ASD or PTSD as a distinct diagnosis, requiring careful and comprehensive clinical evaluation.

The analyzed factors, which were selected based on personal experiences of research team members (OG is a displaced person herself, MM, KKM, MSS, and PK hosted Ukrainians fleeing the war in their homes) can be divided into four groups: demographic characteristics (age, sex, financial resources), circumstances of the displacement (e.g., traveled distance, how long the journey took, whether the study participants experienced bombardments/shellings, left someone behind in Ukraine, with whom they traveled, did someone they knew died during the Russian invasion), their troublesome experiences in the place of refuge (whether they suffered from discrimination, aggression or the feeling of loneliness), which we thought could correlate with ASD and the severity of its symptoms. The fourth group included previous (pre-2/242022) traumas (combat injuries, forced relocation, domestic violence) and autoimmune diseases (thyroid disease, diabetes, irritable bowel syndrome, rheumatoid arthritis, depression, and anxiety). The questions regarding those variables were aimed at verifying the links between ASD and autoimmune diseases, and the peak-end rule (a psychological heuristic in which people judge an experience largely based on how they felt at its peak, i.e., its most intense point) (20–22) regarding the Ukrainian war trauma.

## Methods

### Acute Stress Disorder Scale and its adaptation

A structured research survey consisting of two parts was used. The first conceived purposefully for this study consisted of questions concerning the four abovementioned groups of independent variables.

The second part of the survey was the Acute Stress Disorder Scale (ASDS), a self-report inventory consisting of 19 items based on criteria for ASD as defined by the DSM-IV. The ASDS has been shown to possess good reliability and validity in previous studies and has good sensitivity (95%) and specificity (83%) for identifying ASD compared with clinical interviews (23). In the present study, the ASDS showed high internal reliability (Cronbach α = 0.831).

The threshold score of 37 (≥9 in the Dissociation and ≥ 28 in Reexperiencing, Avoidance, and Arousal subcriteria) was used as a cutoff for diagnosing ASD (24). Since ASDS serves as a measure of current trauma-related distress distinct from its prognostic significance (25), the total score was used as a measure of symptom severity. In addition, validation studies revealed that an ASDS cutoff score of 56 correctly identified 91% of people who developed subsequent PTSD and 93% who did not. It was therefore adopted as the PTSD prediction threshold (24, 26).

Although the ASDS was updated to meet the DSM-5 ASD diagnostic criteria, and its power as a PTSD predictor has been questioned (27), the 19-item version validated for the Polish language was used (24). It is because Ukrainian and Polish populations are alike in many aspects, and the usage of a diagnostic tool already applied in the Central/Eastern European social context had an advantage for comparative reasons.

The ASDS was further adapted in the following way. First, the team of two Eastern studies experts (KKM and MSS) prepared a draft translation of the survey from Polish to Russian and Ukrainian. Both versions were then edited by a Ukrainian and Russian native speaker, a war refugee from the current conflict (OG), and two other persons, a Russian and a Ukrainian native speaker. Based on the mutual feedback, the KKM, MSS, and OG team prepared a final bilingual survey version. To make sure that both language versions are semantically identical, in the second validation round, eight bilingual Ukrainian people of different sex, background, and education (one living in Ukraine, four immigrants, and three refugees from the current conflict, including one MD, two with a Ph.D.) filled in the survey and compared both versions for possible inconsistencies of meaning. Their comments were implemented in the final version of the survey. The research subjects were offered a choice of Russian or Ukrainian language version. 73.16% of the refugees chose the Ukrainian version, 26.84% preferred the Russian version. Both sets of data were analyzed together.

### Sampling

Researchers approached the participants directly from late March to the end of May 2022 (when the first wave of refugees began to drop). The survey was circulated via newly established focused online groups of refugees and displaced persons (usually one per major city in the host countries). The study invitation stipulated that it was addressed to adults (18 and older) fleeing the war and reaching their place of refuge within the last 30 days. In that manner, we obtained a convenience sample of 637 participants from a number of European countries, but mostly Poland (see Table 1). This type of sampling is not representative; however, regarding the dire circumstances, it was the only one available, with the clear advantage of reaching to participants directly during their displacement.

**Table 1:** The refugees’ troublesome experiences in the host countries (*N* = 637).

Country	Experienced discrimination or disenfranchisement due to background and/or cultural differences		Experienced verbal or physical aggression		Experienced a feeling of loneliness*	
	Yes *n* (%)	No *n* (%)	Yes *n* (%)	No *n* (%)	Yes *n* (%)	No *n* (%)
Poland	33 (10.3%)	286 (89.7%)	15 (4.7%)	304 (95.3%)	194 (60.8%)	125 (39.2%)
Germany	14 (18.4%)	62 (81.6%)	3 (3.9%)	73 (96.1%)	58 (76.3%)	18 (23.7%)
Estonia	7 (21.2%)	26 (78.8%)	0 (0.0%)	33 (100%)	16 (48.5%)	17 (51.5%)
Lithuania	4 (15.4%)	22 (84.6%)	1 (3.8%)	19 (96.2%)	14 (53.8%)	12 (46.2%)
Slovakia	2 (10.0%)	18 (90%)	1 (5.0%)	19 (95%)	10 (50.0%)	10 (50.0%)
Netherlands	5 (21.7%)	18 (78.3%)	0 (0.0%)	23 (100%)	16 (69.6%)	7 (30.4%)
Ukraine	7 (15.2%)	39 (84.4%)	0 (0.0%)	46 (100%)	20 (43.5%)	26 (56.5%)
Latvia	4 (25.0%)	12 (75%)	1 (6.3%)	15 (93.8%)	11 (68.5%)	5 (31.3%)
Other	11 (14.1%)	67 (85.9%)	9 (11.5%)	69 (88.5%)	51 (65.4%)	27 (34.6%)
Total	87 (13.7%)	550 (86.3%)	30 (4.7%)	607 (95.3%)	390 (61.2%)	247 (38.8%)

* test *χ*2 = 18.954, df = 8, *p* < 0.05.

### Ethics approval and consent to participate

The authors assert that all procedures contributing to this work comply with the ethical standards of the relevant national and institutional committees on human experimentation and with the Helsinki Declaration of 1975, as revised in 2008. The Poznan University of Medical Sciences Bioethics Committee approved the survey as a non-experimental type (Decision no. KB-781/22). All participants were informed in two languages about the research aims and gave their informed consent for participation.

### Statistical analysis

Logistic regression was used to predict an above-threshold score on the ASDS (both 37-point diagnostic and 56-point PTSD prognosis score). First, bivariate odds ratios for ASD were calculated for demographic and background variables. Next, to identify variables associated with higher scores on the ASDS, bivariate correlations were calculated between ASDS total score and quantitative variables (age, distance covered, time in travel) and T-test, Kruskal-Wallis H, and Mann–Whitney U tests and effect size tests (Cohen’s d, η^2^, r) were used for nominal and ordinal variables (circumstances of the refuge, e.g., witnessing bombardment, losing one’s home, traveling with children, leaving closed ones behind in Ukraine; existing comorbidities, financial situation, experiences in the host country). All the tests were run using IBM SPSS Statistics v.26 software.

## Results

### Sample

The participants (98% women; *M* = 37.8 y;18–76 y; SD = 9.79) migrated from all over Ukraine to Poland (50.1%), Germany (11.9%), Estonia (5.2%), Lithuania (4.1%), the Netherlands (3.6%), Slovakia (3.1%), Latvia (2.5%); 12.2% migrated to other countries, and 7.2% were Internally Displaced Persons (IDPs) who sought refuge in western Ukraine. Most left their families in Ukraine: 46.5% left their spouses, 11.5% their child/children, 74.3% their parents and/or in-laws, and 54.3% their siblings. Only 3.3% of the sample left no family members behind.

During their refuge, the average distance covered was 1342.7 km (Me = 1,306 km, SD = 601.5 km, IQR = 691 km), and the average time of travel was 6.25 days (Me = 4.0, SD = 7.38, IQR = 5.0). They traveled with children (own – 69.9%, related – 9.9% or unrelated – 5.2%), the older adults (16.2%), the disabled (4.9%). Only 12.7% of the studied sample traveled on their own. The majority of participants (76.3%) experienced some form of direct physical threat or mental harm because of the Russian invasion, such as bombardment or injury (see Table 2 for details).

**Table 2:** The refugees’ potentially traumatic experiences (*N* = 637).

	*n*	%
Bombardment/shelling/rocket attack	419	65.8%
My house was destroyed	45	7.1%
I was injured	3	0.5%
Someone I know was hurt	129	20.3%
Someone I knew died	176	27.6%
None of the above	151	23.7%

The refugees were also asked about their pre-24/02 trauma and health status. 6.6% declared to have had a combat operation injury, 5.3% experienced forced relocation, and 10.5% experienced domestic violence. 9.8% of the studied sample suffered from depression and anxiety, 13% from thyroid disease, 9.3% from irritable bowel syndrome, and 4.6% from rheumatoid arthritis.

The studied sample was also asked about their experiences in the countries where they sought refuge. Their financial resources in the host countries were insufficient. Only 15.5% declared that they could survive without any help for at least 3 months (see Table 3). 13.7% suffered from discrimination or disenfranchisement due to their background and/or cultural differences, 4.7% from physical or verbal aggression, and as much as 61.2% experienced a feeling of loneliness (see Table 1). However, the latter depended on the country they went to, with the highest number of lonely refugees in Germany (76.3%), and the lowest in Ukraine (43.5%).

**Table 3:** The refugees’ financial situation at the host country (*N* = 637).

	*n*	%
I do not have money to buy food or pay the rent, I rely on charity	91	14.3%
I have money to buy food for some time, but I cannot afford to pay the rent even in the cheapest type of accommodation	250	39.2%
I have enough money to buy food and to rent the cheapest flat for some time	197	30.9%
I have enough money to buy food and to rent a flat for at least 3 months	99	15.5%

### ASDS scores

**Figure 1:**
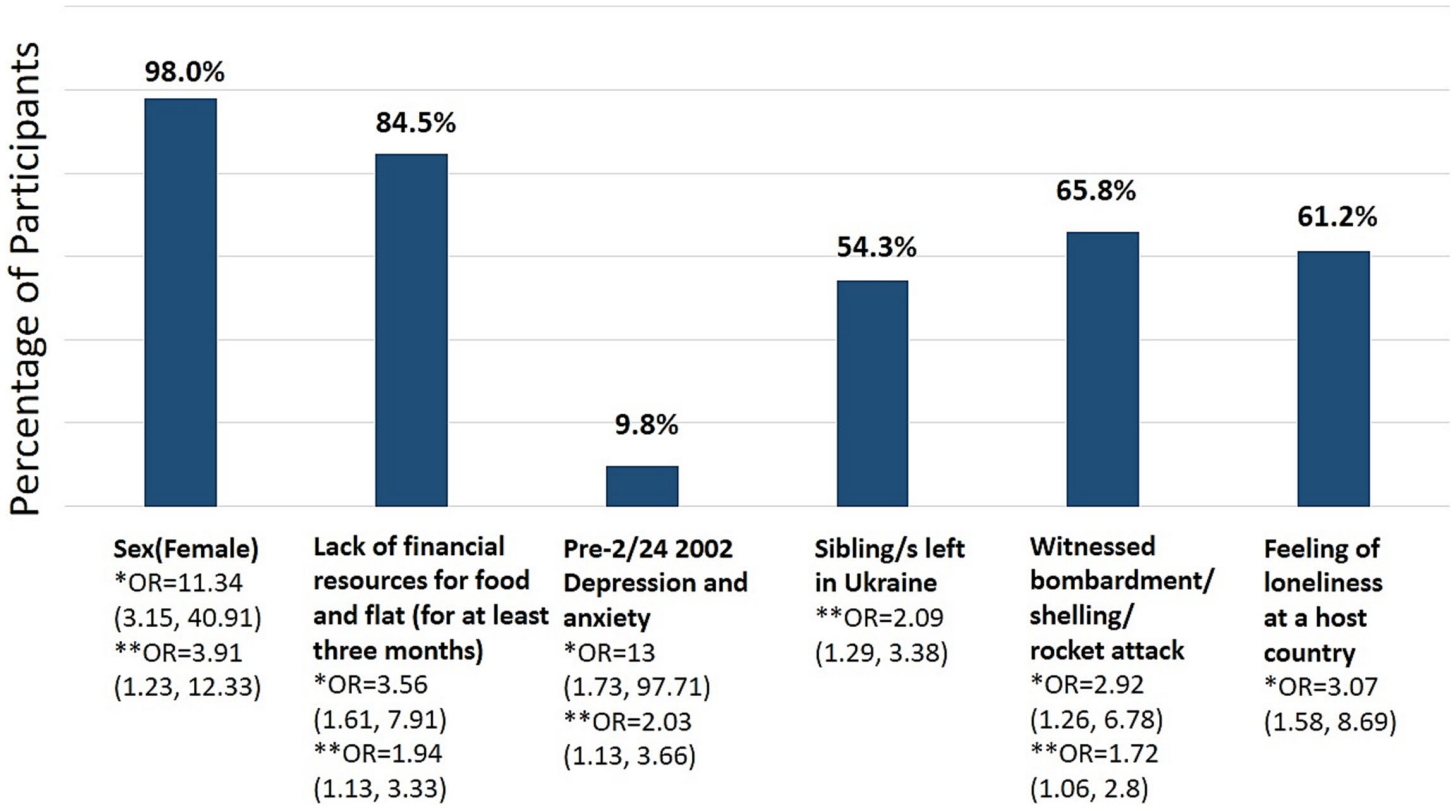
Variables increasing risks of ASD and PTSD development (*N* = 637). Bivariate odds ratios (OR) for associated ASD *(37 point threshold) and subsequent PTSD **(56 point threshold), CI 95%.

ASDS scores ranged from 26 to 94 (mean 66.447, Me 67.0, SD 11.25, IQR 14.0), with 95.3% of the total sample meeting ASD threshold criterion. 85.6% reached the 56-point threshold predictive of the subsequent development of PTSD. The analysis showed no significant differences in ASDS scores between IDPs and those who fled the war abroad (*p* > 0.05). Logistic regression calculated separately for each demographic variable (sex, age), previous (before 2/242022) traumas and diseases, and circumstances of the refuge and their experiences in the host country, revealed variables predictive of scores above both thresholds, that is ASD and PTSD (See Figure 1).

As Figure 1 shows, being a woman increases the likelihood of developing ASD and subsequent PTSD the most. Also, suffering from a prior (pre-war) depression, witnessing bombardment or related attack, the feeling of loneliness in a host country, and the lack of adequate financial resources increases the chances of both ASD and likely the subsequent PTSD while leaving one’s siblings in Ukraine (and neither spouses, parents, nor children) only the PTSD. Interestingly, neither age, the distance covered, time spent on fleeing the country, nor the type of companionship during refuge (escaping alone, with children, pets, or the older adults) correlate with the ASDS scores in the refugee cases that met the ASD diagnostic threshold (*N* = 607). On the other hand, several variables correlate with higher ASDS scores. Refugees who suffered from depression and anxiety before the Russian invasion, left their spouses in Ukraine, witnessed Russian attacks, lost their homes and saw injury and death of others, and were victims of discrimination in the host country suffer from more severe acute stress; however, the effect size of those variables is very small (see Table 4 for details).

**Table 4:** Variables correlating with the severity of ASD symptoms (*N* = 607).

Variable		*n* (%)	Mean (Me; SD) ASD total score	Test results	
Depression and anxiety diagnosed before 2/242022	yes	189 (31.1%)	**71.132 (71; 10.20)**	U Mann–Whitney = 27,601; *r* = 0.24; *η*^2^ = 0.05	*p* < 0.001
	no	418 (68.9%)	66.153 (66; 9.19)		
Spouse left in Ukraine	yes	284 (46.8%)	**68.722 (68; 10.07)**	T-Test = 2.4; Cohen’s *d = 0.*19595	*p* < 0.05
	no	323 (53.2%)	66.808 (67; 9.46)		
Witnessed bombardment/ shelling/ rocket attack	yes	405 (66.7%)	**68.363 (68; 9.85)**	T-Test = 2.36; Cohen’s *d = 0*.20437	*p* < 0.05
	no	202 (33.3%)	66.381 (66; 9.54)		
My house was destroyed	yes	45 (7.4%)	**70.911 (71; 8.78)**	U Mann–Whitney = 10005.000; *r* = 0.095; *η*^2^ = 0.009	*p* < 0.05
	no	562 (92.6%)	67.447 (67; 9.82)		
Someone I know was hurt	yes	126 (20.8%)	**70.913 (73; 9.99)**	U Mann–Whitney = 22980.500; *r* = 0.17; η^2^ = 0.03	*p* < 0.001
	no	481 (79.2%)	66.863 (67; 9.57)		
Someone I knew died	yes	172 (28.3%)	**69.860 (70;10.39)**	U Mann–Whitney =30818.500; *r* = 0.14; η^2^ = 0.019	*p* < 0.001
	no	435 (71.7%)	66.851 (67; 9.41)		
Experienced discrimination or disenfranchisement due to your background and/or cultural differences in the host country	yes	85 (14%)	**69.941 (70; 9.40)**	U Mann–Whitney = 18837.000; *r* = 0.09; *η*^2^ = 0.008	*p* < 0.05
	no	522 (86%)	67.339 (67.5; 9.81)		

The values has been bolded to highlight them as the most important numerical data.

## Discussion

The prevalence of ASD among Ukrainian war refugees was catastrophic. 95.3% of the total sample met the ASD threshold criterion, and 85.6% reached the 56-point threshold predictive of the subsequent development of PTSD. DSM-5 estimates that ASD prevalence among those who experience an interpersonal traumatic event, such as mugging or sexual assault, is as high as 50%, and for other traumatic and catastrophic events less than 20% (28). Nevertheless, evidence indicates that using the DSM-5 criteria results in lower rates of ASD than the DSM-IV criteria [e.g., 14.2 vs. 18.6% (29)] upon which the ASDS is based. Still, the numbers would likely be comparably high. War refugee trauma appears more traumatizing than other stressful events. This shows that the countries hosting the refugees and displaced persons should consider that nearly all will need prompt psychological support regardless of their characteristics. Fewer refugees will require long-term psychological help. Studies show that between 20 and 43% of refugees develop PTSD symptoms (30–34), the most recent study on Syrian refugees in Greece shows that this number can reach 72% of male refugees, who seem more vulnerable than females (35). The mental health problems contribute to other serious health risks those leaving Ukraine face due to the ongoing war: cardiovascular diseases, chronic respiratory diseases, diabetes, chronic infectious diseases, and mental health disorders. At the same time, the Ukrainian population has a very low immunization rate for childhood diseases and Covid-19 (36).

Notably, the declared pre-war history of major depression and/or anxiety disorder was strongly positively correlated with the risk of developing ASD/PTSD. The results support the thesis that mental disorders rarely appear in isolation, and susceptibility to one type of disorder may provide some valuable clinical information about further vulnerability. The results also point to the characteristics of refugees that make them even more prone to develop ASD and suffer from more severe symptoms. Like in the case of Hurricane Katrina victims (37), female refugees and people with fewer financial resources are more vulnerable. Witnessing death and violence, losing home, and leaving loved ones behind also contributes to ASD in Ukrainian refugees in addition to preexisting (pre 02/242022) depression and anxiety. In contrast, male refugees with good financial resources, without comorbidities, and who did not witness bombardment nor feel lonely, are less likely to develop ASD. These results seem to match the findings of previous research in Syrian refugee population, which showed that women, persons with insufficient financial resources, experiencing language barriers, social exclusion, and insufficient emotional support are more vulnerable and suffer from PTSD, anxiety, depression, and low subjective well-being more often (38–40).

Therefore, the peak-end rule was only partially supported by the results. The peak negative experiences (spouse left in Ukraine, witnessing bombardment, home destruction or the death of an acquaintance) were the most important factors influencing the risk of ASD/PTSD development. However, the end experience seems to be of lesser importance due to the vagueness of the causal relation between ASD/PTSD and some other factors; for example, the feeling of loneliness in the host country could potentially be the result of poor mental health as well as a causal factor increasing the severity of ASD. Still, most refugees feel lonely (61.2%), even if moving within Ukraine (43.5%), and they develop ASD three times more often. In contrast to the abovementioned factors, the feeling of loneliness can be addressed in host countries by providing the refugees companionship and support. The same is true of discrimination and disenfranchisement due to background and/or cultural differences reported by 13.7% of the sample, which significantly increases the severity of ASD symptoms. Such discrimination may originate in previous labor migration from Ukraine, which had caused biased attitudes of the local populations, but in contrast to previous war experiences, it may still be subject to change. However, it remains unclear whether discrimination was a trigger augmenting the symptoms of ASD or the result of the hyper-pessimistic expectations about life in the target country.

The research shows no clearly detectable link between the development of ASD and declared autoimmune disorders such as thyroid autoimmune disorders, diabetes, irritable bowel syndrome, and rheumatoid arthritis. However, the study is based on subjective reports only, and the sample is relatively small for measuring such correlation; previous studies confirming the correlation had over 600 k and over 100 k participants, respectively (17, 18). Secondly, only a handful of autoimmune disorders were assessed, whereas other large studies took into account 41 of the most common among them (17).

After the 2013 Revolution of Dignity, Ukraine has been in the most active phase of systemic transformation since 1991. It has manifested itself on several levels: in foreign policy through the signing of an association agreement with the European Union in 2014; in domestic policy, in institutional reforms concerning agriculture, local government, and force structures (41) as well as digitization (42), and in the development of the service sector. Finally, the transformation has manifested itself in a broad change in the sphere of values and memory. The latter involved, among other things, a turn in the public debate about the Ukrainian nation’s past (43). At the same time, phenomena indicative of state dysfunctionality, such as demographic collapse, corruption (44), the inefficiency of the judicial system (45), and the privatization of violence, extended significantly in the border regions of the armed conflict in the Donbas (46), and the influence of big business on legislative bodies persisted (47–49). When reflecting on the condition of Ukrainian society in the near future, it is relevant to take into account the demonstrated scale of acute stress reactions among war refugees. In addition to the challenges it poses to health and welfare systems in the hosting countries, it also generates social problems in Ukraine. Weakened by the acute stress response and its long-term consequences, the population may be less able to help those fighting and loved ones who have remained on Ukrainian territory. Once the war is over, the long-term effects of war-induced stress would likely negatively affect the enthusiasm to continue modernization in the spirit of civic democracy and Europeanization.

## Conclusion

When reflecting on the condition of Ukrainian society in the near future, it is relevant to take into account the demonstrated scale of acute stress reactions among Ukrainian war refugees. In addition to the challenges it poses to health and welfare systems in the hosting countries, it also generates social problems in Ukraine. Weakened by the acute stress response and its long-term consequences, the population may be less able to help those fighting and loved ones who have remained on Ukrainian territory. Once the war is over, in turn, the long-term effects of war-induced stress would likely negatively affect the enthusiasm to continue modernization processes in the spirit of civic democracy and Europeanization.

## Limitations

The major limitation of this study is the study type and sample characteristics. However, considering the circumstances, other types of studies and sampling methods were unavailable. The sample consists primarily (98%) of women, who were the vast majority of Ukrainian refugees (men between 18 and 60 years were officialy not allowed to leave Ukraine).

Using the 19-item ASDS may also be considered a limitation since there is an updated version of the scale (the latter, nevertheless, not available in Polish).

Despite a large sample size (*N* = 637), the study’s results cannot be easily generalized. Since the continuous variables did not present a normal distribution, the authors used mainly nonparametric tests to analyze covariations between the categorical variables and ASDS scores.

To summarize, this study was an attempt to assess the prevalence of ASD among refugees and identify the characteristics of the most vulnerable, but given the resources available and the circumstances in which it took place, it should be treated as an exploratory study.

## Data availability statement

The raw data supporting the conclusions of this article will be made available by the authors, without undue reservation.

## Ethics statement

The studies involving humans were approved by Poznan University of Medical Sciences Bioethics Committee. The studies were conducted in accordance with the local legislation and institutional requirements. The ethics committee/institutional review board waived the requirement of written informed consent for participation from the participants or the participants’ legal guardians/next of kin because the participants were asked to fill in a survey. In the invitation the authors explained the goals of the study and stated that participation is voluntary. Filling in the survey was understood as consent for participation.

## Author contributions

PK: Conceptualization, Formal analysis, Methodology, Writing – original draft, Writing – review & editing. MR: Writing – original draft. MS-S: Conceptualization, Data curation, Investigation, Validation, Writing – original draft. KK-M: Data curation, Resources, Validation, Writing – original draft. OG: Data curation, Validation, Writing – original draft. MM: Conceptualization, Methodology, Supervision, Writing – review & editing.
